# Artificial intelligence in managing retinal disease—current concepts and relevant aspects for health care providers

**DOI:** 10.1007/s10354-024-01069-1

**Published:** 2025-02-24

**Authors:** Sophie Riedl, Klaudia Birner, Ursula Schmidt-Erfurth

**Affiliations:** Department of Ophthalmology and Optometry, Laboratory of Ophthalmic Image Analysis, Medical University of Vienna, Währinger Gürtel 18–20, 1090 Vienna, Austria

**Keywords:** Optical coherence tomography, Artificial intelligence, Retinal disease, Imaging

## Abstract

Given how the diagnosis and management of many ocular and, most specifically, retinal diseases heavily rely on various imaging modalities, the introduction of artificial intelligence (AI) into this field has been a logical, inevitable, and successful development in recent decades. The field of retinal diseases has practically become a showcase for the use of AI in medicine. In this article, after providing a short overview of the most relevant retinal diseases and their socioeconomic impact, we highlight various aspects of how AI can be applied in research, diagnosis, and disease management and how this is expected to alter patient flows, affecting also health care professionals beyond ophthalmologists.

## Retinal diseases

### Socioeconomic impact

Global trends reveal that the numbers of patients affected by vision impairment and blindness are growing and are estimated to increase during the next 30 years [1]. Retinal diseases are the third most common reason for vision loss, following refractive errors and cataract [1, 2]. Globally, age-related macular degeneration (AMD) and diabetic retinopathy (DR) are most prevalent among retinal diseases, with AMD being the most common cause of irreversible vision loss in industrialized countries [2–4].

Novel treatment strategies and continued technological advances have revolutionized retinal care in recent decades. Diagnostically, first and foremost, optical coherence tomography (OCT), offering non-invasive, cross-sectional, three-dimensional display of retinal tissue, has changed the management of numerous retinal diseases and is the imaging modality most commonly applied to the retina [5–7]. Therapeutically, intravitreal injections with anti-vascular endothelial growth factor (anti-VEGF) have opened up a novel therapeutic universe for several relevant exudative retinal diseases, including AMD, DR, and retinal vein occlusion (RVO) since their regulatory approval in 2006 [8–10]. Despite these groundbreaking advances, the chronic progressive nature of these conditions, requiring regular monitoring visits with re-injections every 4, 8, or 12 weeks, in addition to demographic shifts of rising patient numbers in a growing elderly population, lead to a high treatment burden for patients and health care systems [3, 11]. Additionally, in a recent breakthrough, novel treatment based on complement inhibition was approved for non-exudative AMD in the United States, which affects approximately 85% of AMD patients and might increase the treatment burden even further, as no treatment was available for this patient cohort until 2023 [12–14].

## Overview of the most prevalent retinal diseases

### Degenerative

#### Age-related macular degeneration

AMD is estimated to affect 288 million patients worldwide in 2040 [3]. It is a multifactorial disease with genetics, age, smoking, and systemic disease as contributing factors for development and progression [15]. The pathophysiology behind AMD is not fully understood. The deposition of extracellular material and metabolic and vascular alterations due to chronic oxidative stress are believed to play a role [15, 16]. Drusen are the hallmark sign of AMD and histologically represent extracellular lipoprotein deposits located below the retinal pigment epithelium (RPE) [17]. Early and intermediate AMD (iAMD) stages are subclassified based on drusen size and so-called pigmentary changes of the macula and might progress to advanced AMD stages [17]. Patients with early and iAMD are usually asymptomatic, whereas mild symptoms including impairment of twilight vision and metamorphopsia occur in some cases [17]. Late AMD is categorized into neovascular (“wet”) AMD (nAMD) and non-exudative AMD, termed geographic atrophy (GA). Onset of nAMD presents with metamorphopsia, acute or subacute blurry vision, and scotoma, while GA is characterized by chronically enlarging lesions of atrophy, which lead to irreversible loss of visual acuity once the foveal center point is affected. GA lesion growth and the onset of vision-threatening symptoms show high interpatient variability [12, 17]. Early classifications of AMD were based on color fundus photography (CFP), while more recent nomenclature focuses on OCT imaging, which offers far more detailed visualization of pathologic features in all stages of the disease [18, 19]. In OCT, drusen, which are pathognomonic for early and iAMD, are elevations of the RPE. In nAMD, pathologic macular neovascularization (MNV) causes accumulation of fluid in the subretinal space (SRF) and intraretinal space (IRF). The vascular membrane corresponding to the MNV can be displayed in non-invasive OCT angiography (OCT-A) [18]. The underlying chronic progressive course of AMD leads to thinning and atrophy of outer retinal layers, which in its late stage presents as GA [19]. This process can readily be imaged by fundus autofluorescence (FAF) imaging and, again in more detail, by OCT [20]. Currently, there is no available treatment for early and iAMD. Initially, drastic vision loss in nAMD can be prevented by initiation of treatment with anti-VEGF intravitreal injections [21]. However, the chronic course of nAMD requires regular OCT monitoring and monthly, bimonthly, or quarterly reinjection, and long-term functional outcomes may still be unsatisfactory due to chronic disease progression, often leading to atrophy and scarring despite ongoing anti-VEGF therapy [22]. For GA, two novel therapeutics have been approved in the US since 2023 and are administered as monthly or bimonthly injections with the aim of slowing disease progression [13, 14].

### Vascular diseases

#### Diabetic retinopathy

The global population of people affected by diabetes mellitus (DM) is estimated to reach 700 million patients in 2045 [23]. In DM, microangiopathy of retinal capillaries leads to ischemia and compensatory production of proangiogenic stimulators and inhibitors, including VEGF [24]. Beside metabolic glucose control and systemic co-morbidities, disease duration remains a major risk factor for the development of DR [25]. In type 1 DM, 80% of patients will suffer from DR after 15 years of disease duration [26], while 84% of insulin-dependent type 2 DM patients suffer from DR after 19 years [27]. DR is the leading cause of new cases of legal blindness among working-age populations [28]. DR is classified into non-proliferative diabetic retinopathy (NPDR) and proliferative diabetic retinopathy (PDR) [28, 29]. Patients with NPDR are usually asymptomatic, whereas PDR is associated with severe vision impairment, as nearly half of PDR patients will suffer from retinal detachment or vitreous hemorrhage if left untreated [25, 30]. NPDR is further subclassified into mild, moderate, and severe based on the extent of microaneurysms, intraretinal hemorrhages, cotton wool spots, and venous alterations visible throughout the retina. PDR is characterized based on pathologic neovascularization at the optic disc and/or elsewhere in the retinal periphery [31, 32]. These can be diagnosed based on their pathognomonic leakage in dye-based fluorescein angiography, while non-invasive display with single-shot widefield OCT‑A devices is a promising future direction [29, 33]. Ischemia-triggered neovascularization is treated with panretinal laser coagulation and/or intravitreal anti-VEGF injections. Diabetic macular edema (DME), which can occur during any disease stage, clinically presents with metamorphopsia and blurry vision once the fovea is affected and can easily be diagnosed with OCT imaging [25]. Intravitreal anti-VEGF is the gold standard treatment of DME [29].

#### Vascular occlusion

Risk factors for retinal vascular occlusion include systemic diseases such as high blood pressure and DM [34]. Chronic vascular alterations are a risk factor for arterial and venous occlusion, as systemic hypertension results in characteristic changes of retinal capillaries, including the vasoconstriction of arteries and arteriovenous crossing signs detectable in clinical examination. Severe hypertensive retinopathy, a disease entity in its own right, presents with retinal hemorrhages, hard exudates, retinal ischemia, and cotton wool spots. Gold standard treatment of hypertensive retinopathy is management of systemic blood pressure [34].

Retinal vein occlusion can be subclassified into central retinal vein occlusion (CRVO) and branch retinal vein occlusion (BRVO). CRVO presents with acute unilateral blurred vision and macular edema, vessel tortuositas, optic disc edema, and flame-shaped hemorrhages across the whole retina in fundoscopy [35]. BRVO is more common than CRVO. The global prevalence of BRVO is estimated at 0.4% and that of CRVO at 0.08% [36]. OCT imaging characteristically shows central macular edema (CME) [35]. BRVO shows similar clinical findings restricted to the affected areas. Therefore, visual impairment resulting from BRVO might be less pronounced. The gold standard treatment of CME in both CRVO and BRVO is intravitreal anti-VEGF therapy [37]. Complications of RVO include peripheral neovascularization, vitreous hemorrhage, and secondary glaucoma due to rubeosis iridis [35].

Retinal artery occlusion is similarly classified based on the affected region into central retinal artery occlusion (CRAO) and branch retinal artery occlusion (BRAO). Patients with CRAO are affected by complete, painless vision loss, while BRAO patients present with vision loss within the quadrant of the affected artery [38]. Vision impairment due to artery occlusion is irreversible, despite some discussed treatment strategies (bulbus massage, paracentesis, acetazolamide infusion). Systemic workup with neurologic consultation remains essential, as patients with CRAO are at a high risk of future cardiovascular and cerebrovascular events [39, 40].

## Retinal imaging: where AI comes into play

Within the last century, the use of retinal imaging to diagnose and manage the retinal diseases outlined above has become indispensable. In particular, the most recent decades were marked by rapid improvements in retinal imaging techniques, which have sparked not only the application of supporting, AI-based techniques, but also therapeutic developments, as outlined above. While the first images of the living retina, achieved by Jackman and Webster in 1886, depicted blurry, hardly discernible black and white structures [41], the modern range of equipment includes various modalities of two-dimensional retinal imaging, such as CFP, offering documentation of retinal changes in a continuously increasing field of view [42]; fundus autofluorescence [43], predominantly suitable for visualization of RPE-related changes; and dye-based retinal angiography, revealing vascular changes. While some cases may require multiple imaging modalities to be used in a complementary manner with respect to the strengths and weaknesses of the respective techniques, the most frequently applied method, unquestionably, is optical coherence tomography (OCT) [7, 44]. Due to the continued developments of this technique, the groundwork of which was set more than 30 years ago in Vienna [5], modern devices offer three-dimensional cross-sectional imaging of the retina up to an axial resolution of 5 µm within seconds [7]. Further advancement of the technique, termed OCT‑A, allows the indirect, non-invasive visualization of retinal vessels by extracting the information of OCT scans performed sequentially at the same location [45].

As outlined in the previous section, both degenerative and certain vascular retinal diseases show pathognomonic features in OCT imaging and—in the case of DR—on (widefield) CFP. Accordingly, many treatment decisions in routine clinical practice are based on retinal imaging. When asked about their typical approach for patients requiring chronic anti-VEGF treatment, 39.6% of US retina specialists in the Global Trends in Retina Survey 2020, conducted by the American Society of Retina Specialists (ASRS), stated that they acquire an OCT only, without examining the eye at every visit [46]. The resulting immense amount of imaging data presents a platform suitable for the application of AI and the development of AI-based decision-support systems, making retinal care a showcase for the use of AI in medicine [47]. Pioneering work, foremost with respect to AMD, has been performed at the Ophthalmic Image Analysis Laboratory at the University Clinic of Ophthalmology and Optometry at the Medical University of Vienna. The next chapter provides an overview of these and other advances of AI in the field of retinal care.

## What has AI achieved?

### Age-related macular degeneration

Early and iAMD most commonly do not cause patients any symptoms [48]. Clinical examination and multimodal imaging, however, may reveal a disproportionate level of pathologic changes, some of which raise clinicians’ attention with respect to specific risks of progression [44, 49, 50]. Expectations of AI to fulfill the need of identification of high-risk patients are high in light of the therapeutic options emerging for the far more common atrophic late stage of AMD. Robust, commercially available tools for image-based comprehensive risk assessment are still lacking. Nevertheless, AI-based approaches, specifically with respect to image biomarker segmentation, have promoted insight with respect to disease progression. Detection, localization, and quantification of several pathomorphologic features linked to disease progression, such as drusen, thinning of various outer retinal layers, hyperreflective foci, subretinal drusenoid deposits, and choroidal hypertransmission, has been achieved by AI [51–56]. In a further step, feature quantification promotes understanding of disease progression, as temporal changes can be assessed on a large scale, which would not be feasible based on manual image grading. And lastly, the methods which are applied for prediction modeling themselves are continuously being advanced by AI. Vienna’s Ophthalmic Image Analysis Group (OPTIMA) has performed several pioneering investigations in all of these fields. By applying AI-based image segmentation, Riedl et al. identified topographically colocalized, subclinical photoreceptor thinning to be present even more than a year before the occurrence of an early atrophic marker, i.e., outer plexiform layer (OPL) subsidence (Riedl et al. IOVS in press) [57]. Furthermore, machine learning (ML)-based models have been developed for both topographically constricted changes such as predicting a known precursor of disease progression, namely drusen regression, by incorporating various segmentation-derived imaging features and achieving an accuracy of 0.75 [51] but have also been employed at a higher eye-based level for predicting the development of both neovascular and late atrophic AMD by including demographic, genetic, and OCT-based imaging features [58]. Achieving a high accuracy of an area under the curve (AUC) of 0.8 for the prediction of GA and 0.68 for nAMD, features differed notably in the ranked importance between the two tasks. These results highlight the capability of AI-guided analyses to detect relevant trends with respect to disease progression. Moreover, innovative AI-based, self-supervised learning (SSL) methods, such as Morph-SSL, enabling the simulation of OCT transformation between different timepoints have been applied by the Vienna group in the field of AMD and are about to advance knowledge of the risk of individual progression even further [59]. Most recently, a large prospective multicenter study (I-Screen) supported by the EU HORIZON program has started that will perform community-based OCT screening of individuals with iAMD and identify risk profiles which shall ultimately be detected by fully automated screening tools [60].

**Fig. 1:**
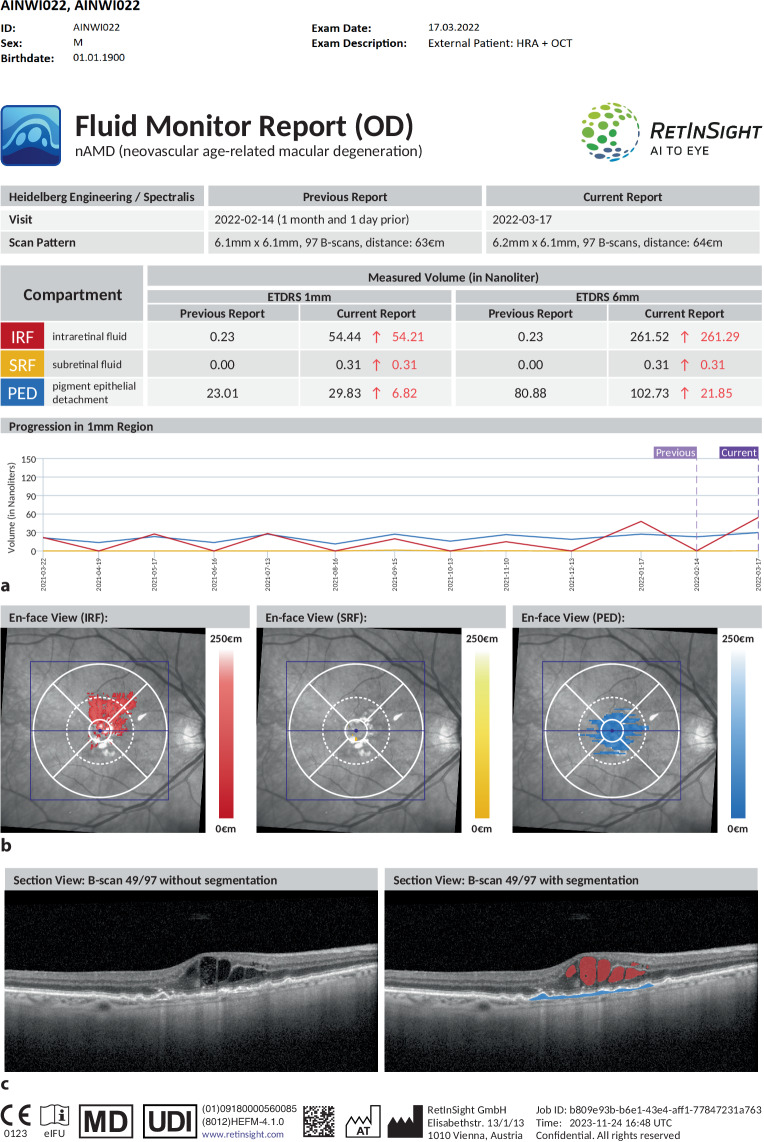
Example Fluid Monitor Report, as provided by RetInSight (Vienna, Austria), indicating volumes of intraretinal fluid (*IRF*), subretinal fluid (*SRF*), and pigment epithelium detachment (*PED*) at current and previous visits in nanoliters (**a**); **b** en-face visualization of fluid quantification; **c** central B‑scan with and without automated feature segmentation

As previously outlined, nAMD is a chronic disease requiring long-term invasive treatment. While fixed treatment regimens can be applied, most clinicians prefer a flexible treat-and-extend regimen, in which re-treatment intervals are determined based on OCT imaging [46]. IRF and SRF as well as the configuration of pigment epithelial detachment (PED) constitute signs of disease activity with certain functional implications and can readily be assessed by automated image segmentation [61]. The use of AI for image feature quantification and prediction modeling has revolutionized understanding and management of the disease, and (re)-treatment guided by AI-based image analysis is about to enter clinical practice [62]. In 2022, the first AI algorithm for evaluation of OCT images was approved as a decision-support system according to MDR for the treatment of neovascular AMD in the European Union (Fluid Monitor, RetInSight, Vienna, Austria). The Fluid Monitor allows an upload of routine OCT images to a cloud and provides fully automated fluid detection, visualization, and quantification in real time. An example report, such as it would be directly obtained by the physician, is shown in Fig. 1.

**Fig. 2:**
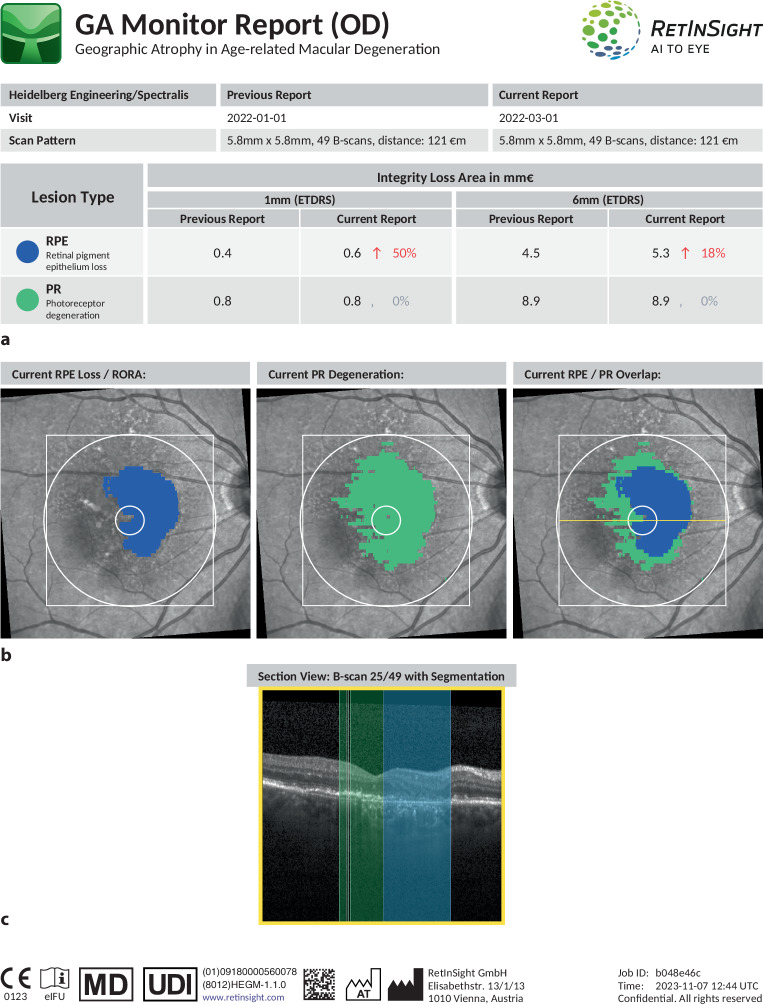
Example GA Monitor Report, as provided by RetInSight (Vienna, Austria), showing automated quantification of retinal pigment epithelium (*RPE*; blue) and ellipsoid zone (*EZ*; green) loss area (mm^2^) numerically (**a**) and in en-face visualization (**b**) as well as the central B‑scan (**c**) including automated segmentation results. EZ loss (mm^2^, green) and RPE loss (mm^2^, blue) are derived from automated segmentation of optical coherence tomography (*OCT*) volumes by deep-learning algorithms and represent OCT regions with loss of these pathognomonic retinal layers. Subclinical loss of the EZ, which is a part of photoreceptors, quantified on OCT imaging precedes loss of RPE, which represents GA area growth

What was a less popular field of AI application has recently become possibly one of the most promising: geographic atrophy. The first treatments for GA are finally available to patients in the US [13, 14], with a negative decision on the use of pegcetacoplan by the regulatory agencies in Europe; therefore, there is currently no available treatment for GA in Europe [63]. Still, with the disease being continuously progressive but largely asymptomatic until advances stages, morphology-based guidance of monitoring and treatment is highly sought. Morphologic changes of GA are identifiable for ophthalmologists on OCT. However, continuous progression of OCT signs, such as RPE loss and particularly photoreceptor thinning and loss, defined as ellipsoid zone (EZ) disappearance, may be subtle and not identifiable in enough detail based on human evaluation in everyday clinical practice. An automated real-time tool has been developed (GA Monitor, RetInSight, Vienna, Austria) which visualizes and quantifies RPE and EZ loss areas in clinical practice (Fig. 2). Such AI-based quantification of various levels of the atrophic lesion, namely the photoreceptors/EZ and the RPE, has enabled at least two groundbreaking innovations. Firstly, robust disease monitoring and progression modeling by offering reproducible quantification of possibly subclinical disease progression in various affected levels of tissue [64]. Secondly, in-depth investigation of the therapeutic effects on morphologic disease progression. Taking these approaches together, AI-based investigations have identified the ratio of photoreceptor loss to RPE loss as a relevant biomarker for both high-risk progressors as well as for patients benefitting most from treatment (Schmidt-Erfurth et al., Ophthalmology in press).

### Diabetic retinopathy: AI for screening

**Fig. 3:**
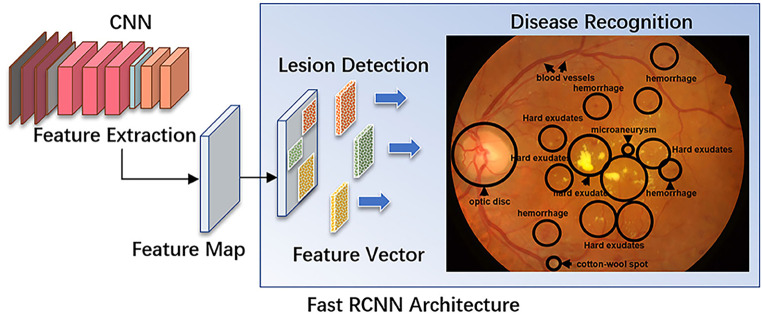
A convolutional neural network (*CNN*) for automated lesion detection and disease recognition of diabetic retinopathy from fundus images. The input image is fed into the CNN network to obtain the corresponding feature map. The derived feature map is used to estimate region proposals (candidate regions in squared boxes), which are then classified and predict different disease categories. *R‑CNN* region-based CNN. Reproduced from [82]

Diagnosis of DR at an early disease stage allows scheduling of personalized monitoring visits and timely treatment to avoid progression to vision-threatening complications [65]. Staging and diagnosis of DR is easily performed based on the above-mentioned findings in non-invasive CFP, a task most suitable for the use of AI (Fig. 3). AI-based detection of DR on CFP for screening purposes has comparable diagnostic accuracy to that of specialists [66]. Therefore, automated screening models were proven to be well suited to ease the disease burden by autologous automated CFP-based screening programs in primary care offices [67]. In 2018, the first AI-based DR screening device, IDx-DR (IDx Technologies Inc., Coralville, IA, USA), was approved by regulatory agencies in the US with the goal of allowing for nationwide screening in primary practice offices [68]. Currently, there are two autonomous AI systems for DR screening without human evaluation that are approved by the FDA: IDx-DR [67] and EyeArt [69, 70]. IDx-DR performs automated detection of DR and DME from CFP with a diagnostic sensitivity of 87.2% and specificity of 90.7% [67]. EyeArt (Eyenuk, Inc., Woodland Hills, CA, USA) reports 96% sensitivity and 88% specificity for detecting DR and DME and additionally provides detection of vision-threatening DR with 97% sensitivity and 90% specificity [69].

### Taking the broader view: what do retinal changes reveal about the body?

Studies have revealed a correlation between alterations of retinal vasculature in CFP and cardiovascular health [71]. Due to its easy accessibility by imaging, the fine retinal microvasculature is a suitable target for screening of cardiovascular disease. The development of automated tools for population-based screening with timely recognition and targeted treatment of common heart disease may greatly relieve health care systems [72]. This is of utmost interest, as cardiovascular pathologies remain one of the major causes of death worldwide, with an increasing number of deaths from heart disease despite diagnostic and therapeutic advances [73]. OCT‑A, the availability of which is becoming increasingly more widespread, offers even more precise insights into retinal microvasculature [74]. Again, due to the easy and non-invasive image acquisition, the resulting data are most suitable for AI-based analysis and the recognition of patterns behind early vascular alterations in hearth disease. Subclinical OCT angiography parameters, such as vessel density and foveal avascular zone measurements, seem promising for future developments [75]. A deep-learning network was recently developed to predict age, systolic blood pressure, body mass index (BMI), and HbA1c from retinal imaging. An additional model was trained to predict major cardiovascular events from retinal imaging (AUC 0.70) [72].

Early detection of central nervous system disorders via OCT‑A represents another promising implementation of non-invasive retinal imaging, with emerging evidence to support this concept. Vascular density has been shown to be significantly reduced in patients with Alzheimer’s disease compared to controls. Moreover, detection of subclinical Alzheimer’s disease with OCT‑A has been achieved in small cohorts. Similar findings based on different OCT‑A parameters were demonstrated in Parkinson’s disease. In multiple sclerosis, biomarkers relating to optic nerve head perfusion seem to be of interest, among other novel features [76].

More recently, a foundation model (RETFound) which learns from unlabeled image data was trained for self-supervised learning for the diagnosis and progression assessment of common retinal diseases [77].

## Future implications for health care providers

Given the outlined advances and expected increase in telemedical services, patient flows are expected to change in the future. While groundbreaking improvements with respect to health care inequality are anticipated for developing countries [78], the authors focus below on implications for industrialized countries.

Firstly, physicians, most likely general practitioners and specialists for internal medicine, will be increasingly exposed to screening for retinal diseases associated with systemic disease, such as diabetes and cardiovascular disease. CFP-based screening for DR, but also OCT-based screening for AMD, may affect non-ophthalmologic health care professionals and hopefully lead to an increase in early diagnosis, timely treatment, and preservation of visual function in these highly prevalent diseases. Automated referral recommendation based on OCT imaging is being developed continuously [79]. Furthermore, the role of retinal vascular imaging in screening for systemic disease is expected to increase. With respect to patients with known retinal disease undergoing long-term treatment, patient-operated OCT devices including built-in automated image evaluation have become available for home monitoring [80]. While relevant changes detected by these devices will prompt patients to consult with their treating ophthalmologist, general practitioners might still be required to be familiarized with basic concepts of the disease.

Secondly, paramedical professions specific to ophthalmology, such as opticians and optometrists, may play an increasingly important role in patient management. When it comes to reducing the burden of tertiary clinics, which will need to perform an immensely increasing number of treatments in the near future due to emerging therapies for late atrophic AMD, mere monitoring of patients with known retinal disease might be a suitable task for distribution among more easily accessible ophthalmology-related professions or lower-threshold ophthalmic institutions. AI-based monitoring of early and iAMD but also of later-stage atrophic AMD, once more-defined guidelines exist with respect to treatment initiation, may represent such a use case.

Precise concepts of altered patient flows will be highly dependable on existing systems of care, which differ substantially between countries, and on the distribution and accessibility of the necessary imaging infrastructure. Furthermore, many challenges, such as the lack of diversity used for algorithmic training [81], remain to be tackled. Nevertheless, while the speed and extent of changes in patient management may remain uncertain, their approach is inevitable and will hopefully lead to an improved pooling of resources from which both patients and health care givers will benefit.
